# P-1913. Implementation and Evaluation of a Curriculum on Antimicrobial Stewardship for Pediatric Residents

**DOI:** 10.1093/ofid/ofaf695.2082

**Published:** 2026-01-11

**Authors:** Matthew M Sattler, Sara Greer, Christine R Lockowitz, Monica Abdelnour, Valerie Yuenger, Alexander S Plattner, Jason G Newland, Evan E Facer, Katie Wolfe

**Affiliations:** Washington University in St. Louis School of Medicine, St. Louis, MO; Washington University in St. Louis School of Medicine, St. Louis, MO; St. Louis Children's Hospital, St. Louis, Missouri; Washington University, St. Louis, Missouri; St. Louis Children's Hospital, St. Louis, Missouri; WashU Medicine / St. Louis Children's Hospital, St Louis, MO; Nationwide Children's Hospital, Columbus, OH; Washington University in St. Louis School of Medicine, St. Louis, MO; Washington University in St. Louis School of Medicine, St. Louis, MO

## Abstract

**Background:**

Previous work has identified that an antimicrobial stewardship (AS) curriculum for pediatric residents should focus on empiric therapy and duration of therapy. The impact of a curriculum built from these findings was evaluated.

**Table 1:**
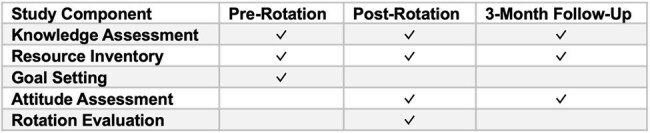
Study Data Collection Timeline Outline of components evaluated on the pre-, post-, and 3-month follow-up assessments.

**Figure:**
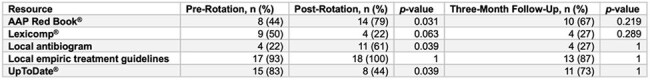
Resource Utilization Utilizing a free-response question, we asked to identify at least three resources they would use to treat a suspected or confirmed infection. We compared the percentage of respondents who identified a specific resource at pre-rotation, post-rotation, and follow-up using McNemar’s test.

**Methods:**

Pediatric residents assigned to an AS rotation from March-August 2024 were eligible to participate. Residents presented patients as part of “handshake stewardship” rounds, completed asynchronous modules developed for the rotation, and participated in weekly mock case discussions. Residents completed assessments on the first day of the rotation (pre-rotation), on the final day of the rotation (post-rotation), and three months following the rotation (follow-up) assessing rotation goals, knowledge, resource utilization, attitudes, and satisfaction (Table 1). Responses were compared using the Wilcoxon signed-rank test and McNemar’s test, as appropriate.

**Figure 1:**
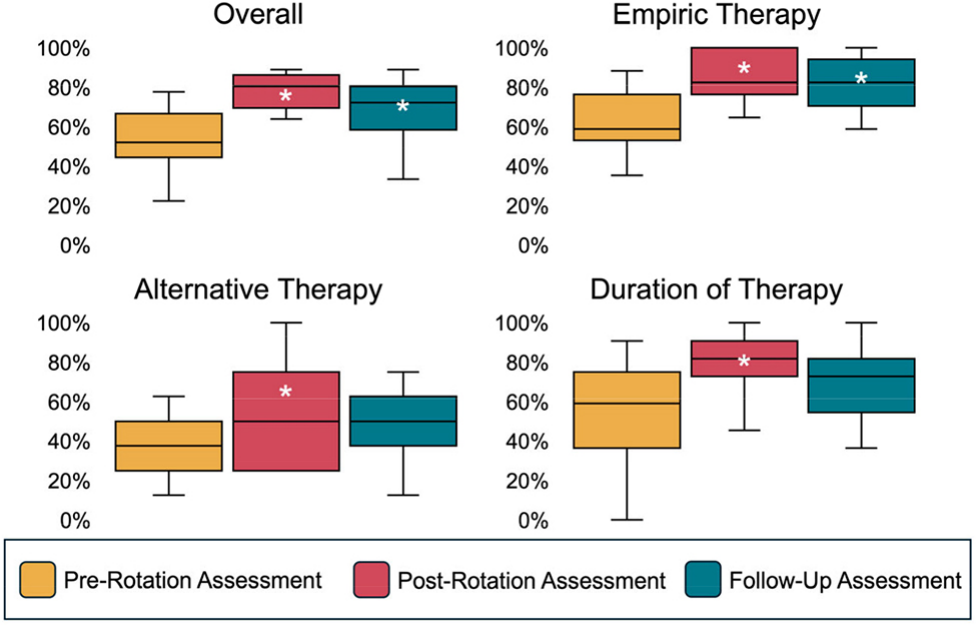
Median Knowledge Assessment Scores A brief clinical scenario was provided, and participants were asked to fill in the blank with the appropriate empiric therapy, alternative therapy (for patients with allergies to first-line therapy), or duration of therapy. Knowledge assessment scores were compared, as well as sub-scores in the domains of empiric antibiotic therapy, alternative antibiotic therapy, and duration of therapy, between pre-rotation, post-rotation, and follow-up assessments using the Wilcoxon signed-rank test. An asterisk indicates a statistically significant difference when compared to the pre-rotation assessment.

**Figure 2:**
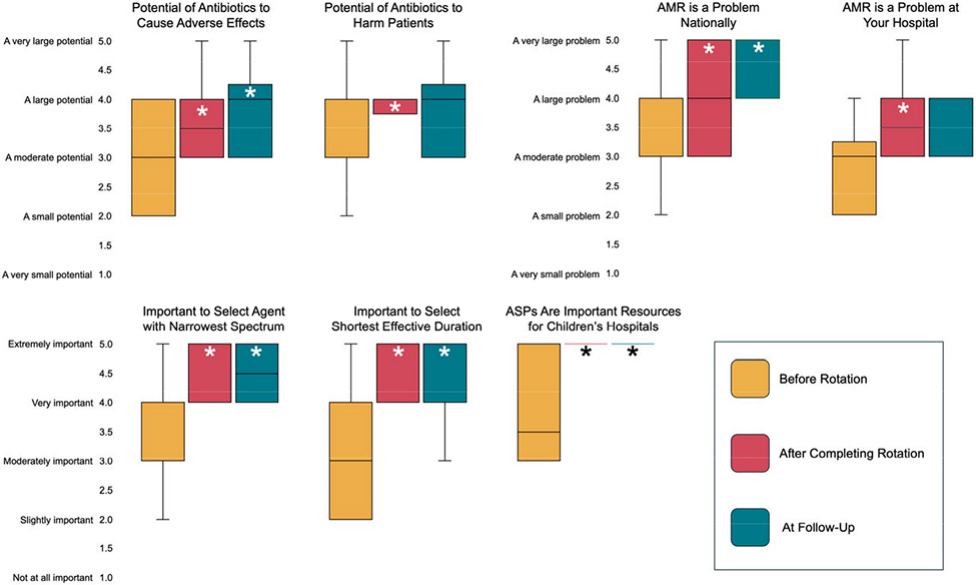
Median Attitude Assessment Responses On the post-rotation assessment, regarding attitudes related to antimicrobial stewardship, we utilized a retrospective pre-/post- methodology to ask participants to consider how they would have responded prior to participating in the rotation, compared to how they would respond now that they have completed the rotation. On the follow-up assessment, we asked respondents to consider their attitude at only that moment in time. We compared the responses using the Wilcoxon signed-rank test. An asterisk indicates a statistically significant difference when compared to before rotation attitudes.

**Results:**

Eighteen participants were enrolled, all of whom completed the pre-/post-rotation assessments; 15/18 (83%) completed the follow-up assessment. Median knowledge assessment scores improved from pre- to post-rotation (50.0% to 77.7%, *p*< 0.001), and remained higher than pre-rotation at follow-up (72.2%, *p*=0.001) (Figure 1). Only the improvement in the empiric therapy sub-score was significant at follow-up. Residents were more likely to identify the AAP Red Book^®^ and the local antibiogram as resources for the treatment of suspected infections immediately after completing the rotation; no significant difference was observed at follow-up (Table 2). Median Likert responses on the attitude assessment improved from before to after the rotation and were sustained at follow-up for 5/7 questions (Figure 2). Nearly all (17/18) rated the quality of the rotation as “excellent.” At follow-up, 64% reported using knowledge from the rotation at least several times per week since completing the rotation.

**Conclusion:**

Participation in a clinically-embedded AS curriculum focused on empiric therapy and duration of therapy was associated with sustained improvements in knowledge and AS-related attitudes. Future work is needed to address recall of high-quality resources and knowledge related to alternative therapy and duration of therapy, as well as to evaluate the potential clinical impact.

**Disclosures:**

Christine R. Lockowitz, PharmD, BCIDP, AbbVie: Grant/Research Support|Premier, Inc.: Honoraria Jason G. Newland, MD, MEd, Pfizer: Grant/Research Support Evan E. Facer, DO, AbbVie, Inc: I have no relevant disclosures, but I receive funding from AbbVie, Inc. for characterization of ceftazidime/avibactam use in children.

